# C-Terminal Truncated HBx Facilitates Oncogenesis by Modulating Cell Cycle and Glucose Metabolism in FXR-Deficient Hepatocellular Carcinoma

**DOI:** 10.3390/ijms24065174

**Published:** 2023-03-08

**Authors:** Xuejun Wu, Zhengzhong Ni, Tiantian Song, Wenya Lv, Yan Chen, Danmei Huang, Yangmin Xie, Weiyi Huang, Yongdong Niu

**Affiliations:** 1 Department of Pharmacology, Shantou University Medical College, Shantou 515041, China; 2 School of Public Health, Shantou University, Shantou 515063, China; 3 Department of Experimental Animal Center, Medical College of Shantou University, Shantou 515041, China

**Keywords:** hepatocellular carcinoma (HCC), C-terminal truncation, hepatitis B virus X protein (HBx), farnesoid X receptor

## Abstract

Farnesoid X receptor (FXR) is a nuclear receptor known to play protective roles in anti-hepatocarcinogenesis and regulation of the basal metabolism of glucose, lipids, and bile acids. FXR expression is low or absent in HBV-associated hepatocarcinogenesis. Full-length HBx and HBx C-terminal truncation are frequently found in clinical HCC samples and play distinct roles in hepatocarcinogenesis by interacting with FXR or FXR signaling. However, the impact of C-terminal truncated HBx on the progression of hepatocarcinogenesis in the absence of FXR is unclear. In this study, we found that one known FXR binding protein, a C-terminal truncated X protein (HBx C40) enhanced obviously and promoted tumor cell proliferation and migration by altering cell cycle distribution and inducing apoptosis in the absence of FXR. HBx C40 enhanced the growth of FXR-deficient tumors in vivo. In addition, RNA-sequencing analysis showed that HBx C40 overexpression could affect energy metabolism. Overexpressed HSPB8 aggravated the metabolic reprogramming induced by down-regulating glucose metabolism-associated hexokinase 2 genes in HBx C40-induced hepatocarcinogenesis. Overall, our study suggests that C-terminal truncated HBx C40 synergizes with FXR deficiency by altering cell cycle distribution as well as disturbing glucose metabolism to promote HCC development.

## 1. Introduction

Hepatocellular carcinoma (HCC) is the predominant subtype of liver cancer, comprising 85%, and is the fifth most common cause of cancer-related death [1]. Chronic hepatitis B virus (HBV) infection has become a substantiated etiologic factor for HCC around the world [2]. HBx is a key regulator in HBV-induced hepatocarcinogenesis, and may act by interacting with host molecules to exert biological functions [3,4,5]. The full-length HBx gene has a length of 465 bp and encodes a 154-amino acid protein. However, partial deletions frequently occur during integration, with C-terminal truncated mutants often being integrated into the human chromosomes [6]. Zhang et al. detected 59 cases of C-terminal truncated HBx in 87 cases of HBV-positive patients (67.8%) and 70% of positive events were confirmed by Amaddeo et al. [7,8]. Multiple truncated HBx proteins, especially C-terminal truncated HBx (ct-HBx) proteins, have been identified to play various roles in HCC [9,10,11,12].

The farnesoid X receptor (FXR), a member of the nuclear receptor subfamily, is highly expressed in the liver and intestine. FXR not only physiologically modulates enterohepatic bile acid synthesis, lipid, and glucose homeostasis, but is also involved in hepatic fibrosis and the pathogenesis of liver cancers [13,14,15]. Compared with normal liver and paracancerous tissues, the expression of FXR or FXR signaling is downregulated in the livers of HCC patients [16]. Previous studies have suggested that aging Fxr-null mice spontaneously develop liver tumors with increased inflammatory cytokines, and cell proliferation by activating the Wnt/β-catenin and c-Myc signaling pathways [17,18,19]. In addition, the expression of FXR is negatively correlated with several clinicopathological characteristics of HCC [16,20]. Our previous study suggested that transactivation of FXR by HBx may play a protective role, and this effect would be diminished by exposure to ct-HBx, especially under FXR deficiency [21]. However, the effect of C-terminal truncated HBx on hepatocarcinogenesis in FXR deficiency has not been elucidated.

Small heat shock protein 8 (HSPB8) is a typical member of the small heat shock protein family [22]. The main role of the family is to trigger cellular stresses, effectively control protein folding and prevent the accumulation of denatured or improperly folded proteins [22]. HSPB8 expression and function in different cancers are different. Previous studies have shown that HSPB8 is involved in cell division, such as cell cycle arrest, regulation of inflammatory responses, and cell proliferation, migration, invasion, and apoptosis [23,24,25,26]. All of the above suggest that HSPB8 may be an essential factor in the development of cancer and a potential target for cancer treatment for specific types of cancer.

In the present study, to investigate the effect of C-terminal truncated HBx on the progression of hepatocarcinogenesis in FXR deficiency and preliminarily analyze its mechanism, we demonstrated that C-terminal truncated X protein (HBx C40) exhibits distinct profiles concerning their regulation of cell growth and hepatocellular carcinoma development in FXR deficiency. This may be through affecting the cell cycle, which further affects glucose and lipid metabolism. It also provides a potential target for exploring the role of truncated HBx in hepatocarcinogenesis and its mechanisms.

## 2. Results

### 2.1. Underexpressed FXR in HBV-Related HCC

**Figure 1:**
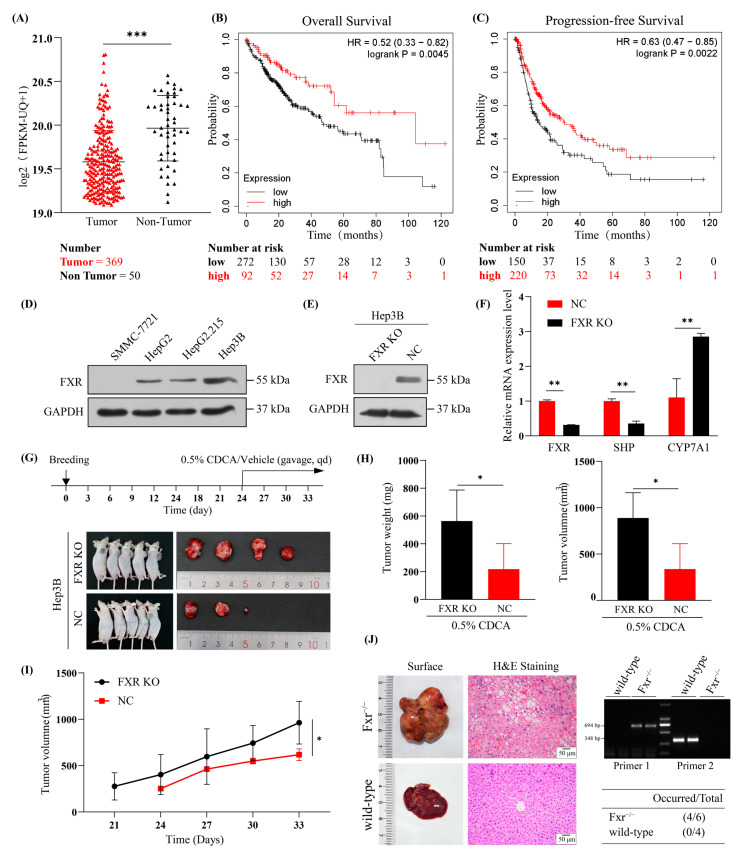
Low FXR expression correlates with poor prognosis in HBV-related HCC and FXR KO promotes liver tumorigenesis. (**A**) The differences in FXR mRNA expression levels between normal (*n* = 50) and liver cancerous tissues (*n* = 369) in TCGA. (**B**,**C**) are Kaplan-Meier analyses of 153 HBV-related HCC patients regarding OS and PFS with high and low FXR expression. (**D**) Expression of FXR in SMMC-7721, HepG2, HepG2.215, and Hep3B cells. (**E**,**F**) Effect of FXR knockout on the expression of downstream FXR target genes of FXR, SHP, and CYP7A1, as examined by western blotting and qRT-PCR. (**G**) Schematic diagram indicating daily gavage of Vehicle or 0.5% CDCA from day 24. NC and FXR KO Hep3B tumorigenesis in vivo (*n* = 5). (**H**,**I**) Statistical plots show each group’s tumor size (mm^3^), tumor weight (mg), and tumor growth (mm^3^). (**J**) Demonstration of hepatic tumors and H&E staining in Fxr^−/−^ and wild-type mice. Scale bar = 50 μm. Genotypes of mice were detected by agarose gel electrophoresis (*n* = 2). A single band of 694 bp represents Fxr^−/−^ mice. A single band of 348 bp represents wild-type mice. The cases of spontaneous tumorigenesis in the total cases of Fxr^−/−^ and wild-type mice. * *p* < 0.05, ** *p* < 0.01, *** *p* < 0.001.

We previously showed that FXR expression is reduced in middle and advanced HBV-associated HCC compared with normal controls or adjacent noncancerous tissues [27]. Underexpression of FXR has been reported to be associated with poor prognosis in HCC [16]. In addition, a comparison between the expression of FXR in normal and tumor tissues in TCGA, by using UCSC XENA (https://xena.ucsc.edu/compare-tissue/, accessed on 20 May 2022), showed that FXR expression was significantly decreased in 369 liver cancer tissues compared to 50 normal tissues (Figure 1A). Kaplan-Meier analysis (https://kmplot.com/analysis/, accessed on 1 April 2022) revealed a significant reduction in overall survival (OS) (Figure 1B) and progression-free survival (PFS) times (Figure 1C) in HBV-related HCC patients with underexpressed FXR.

To investigate the effect on tumorigenesis after FXR KO in vivo, we first determined FXR protein expression in different hepatocellular carcinoma cell lines (Figure 1D) and selected Hep3B cells to construct an FXR knockout hepatocellular carcinoma cell line (FXR KO Hep3B) using the CRISPR/Cas9 system. In FXR KO Hep3B cells (Figure 1E), we showed a decrease in the tendency of small heterodimer partner (SHP, also known as Nr0b2) expression (Figure 1F). Following injection of FXR KO Hep3B cells and corresponding negative control cells (NC) into nude mice (*n* = 5), after treatment with CDCA (an agonist of FXR), tumors appeared earlier in the FXR KO group compared to the NC group (Figure 1G–I). At the same time, the FXR KO group had more tumors and larger tumors than the control group, suggesting that the FXR signaling pathway is disadvantageous to the development of liver tumors. Furthermore, we also observed spontaneous liver tumors in Fxr^−/−^ mice from our laboratory at 15 months (Figure 1J), which differs from our previous data that showed no tumors being formed in the Fxr^−/−^ mice from the Jackson Laboratory [21]. This may be related to the different genetic backgrounds of the mice. The above results all indicate that FXR plays an essential role in the development of HBV-related HCC.

### 2.2. HBx C40 Promotes Cell Proliferation and Migration in FXR KO Hep3B Cells In Vitro

**Figure 2:**
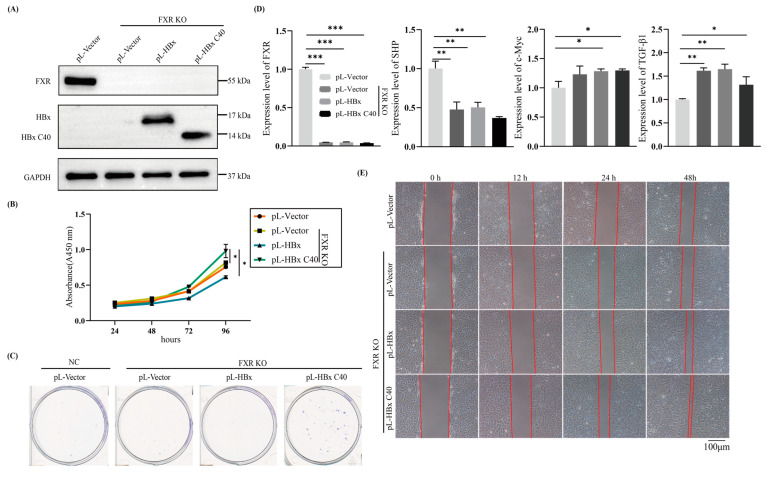
HBx C40 promotes cell proliferation and migration in FXR KO tumor cells in vitro. (**A**) Protein expression levels of FXR, HBx, and HBx C40. (**B**,**C**) Proliferation of FXR KO Hep3B cells following overexpression of HBx or HBx 40 was determined by CCK-8 and colony formation assays. (**D**) The mRNA level expression of FXR, SHP, c-Myc, and TGF-β1 were detected by qRT-PCR. (**E**) Wound healing assays were performed on Hep3B cells in the NC, FXR KO, FXR KO + HBx, and FXR KO + HBx C40 Hep3B cells. * *p* < 0.05, ** *p* < 0.01, *** *p* < 0.001.

Hep3B cells contain an integrated hepatitis B viral genome and continuously express HBx [28]. To define the biological functions of full-length versus C-terminal truncated HBx in FXR deficiency, especially the transactivation of FXR by full-length HBx and the complex relationship between full-length HBx versus C-truncated HBx [21,29], we constructed an FXR knockout Hep3B stable cell line. Then we constructed stable cell lines overexpressing HBx or HBx C40 by using lentiviral vectors to transduce the full-length HBx or HBx C40 genes into FXR KO Hep3B cell lines (Figure 2A). CCK-8 assay, to evaluate the effect of HBx or HBx C40 on the proliferation of NC or FXR KO cells, showed that FXR KO promoted cell proliferation compared to the NC group, and cell proliferation was enhanced by HBx C40 expression compared with the FXR KO control and HBx groups (Figure 2B). Consistent with the CCK-8 results, colony formation assays showed that HBx C40 overexpressing cells formed a greater number of colonies compared to the FXR KO cells, which formed more colonies than wild-type Hep3B cells (Figure 2C). qRT-PCR analysis revealed that HBx C40-mediated enhanced proliferation was related to the upregulation of SHP, c-Myc and TGF-β1 (Figure 2D). In addition, HBx C40 enhanced cell motility in FXR KO cells, as demonstrated by a wound healing assay (Figure 2E).

### 2.3. The Impact of HBx C40 on Cell Cycling and Apoptosis in FXR KO Hep3B Cells

**Figure 3:**
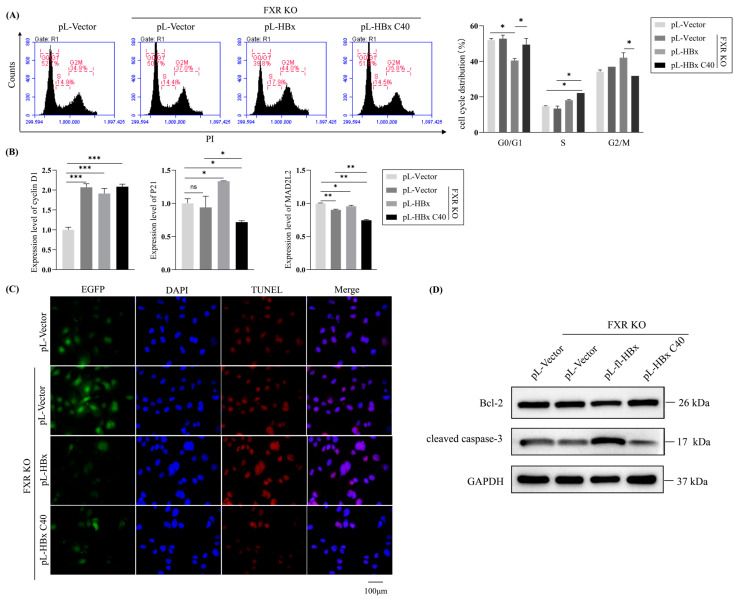
Impact of HBx C40 on the cell cycle and apoptosis in FXR-deficient Hep3B cells. (**A**) Flow cytometry was analyzed to assess the impact of HBx or HBx C40 on the cell cycle in FXR KO Hep3B cells. (**B**) The mRNA expression of cycle-related genes cyclin D1, P21, and MAD2L2. (**C**) TUNEL assay showing the effect of HBx or HBx C40 on apoptosis in FXR KO Hep3B cells. (**D**) Protein expression of Bcl-2 and cleaved caspase-3 after overexpressing HBx or HBx C40 in FXR KO Hep3B cells. * *p* < 0.05, ** *p* < 0.01, *** *p* < 0.001.

To further evaluate the HBx C40-enhanced proliferation and demonstrate a cell-cycle regulatory role for HBx or HBx C40 on the cell cycle in FXR KO Hep3B cells, we examined the effect of HBx or HBx C40 on cell cycle distribution by flow cytometry (Figure 3A). Compared to FXR KO controls, the expression of P21 was upregulated, expression of MAD2L2 was decreased and G2/M phase cell cycle arrest was induced by HBx overexpression, whereas HBx C40 expression downregulated P21 expression, decreased inhibition of cyclin D1 and increased the proportion of cells in the G2/M phase (Figure 3B).

In addition, we also detected the variation in HBx or HBx C40 in apoptosis by TUNEL assay (Figure 3C). More apoptosis was observed in FXR KO Hep3B cells overexpressing HBx, whereas the reverse occurred with overexpression of HBx C40. Furthermore, we checked the expression levels of apoptosis-related regulatory protein Bcl-2 and cleaved caspase-3. HBx suppressed Bcl-2 and elevated cleaved caspase-3 in FXR KO Hep3B cells (Figure 3D). This indicates that the effects of HBx C40 on liver tumor biological behavior correlate with the reduction in cell cycle G2/M and apoptosis.

### 2.4. The Biological Effect of HBx C40 in Transiently Overexpressed Mouse Models

**Figure 4:**
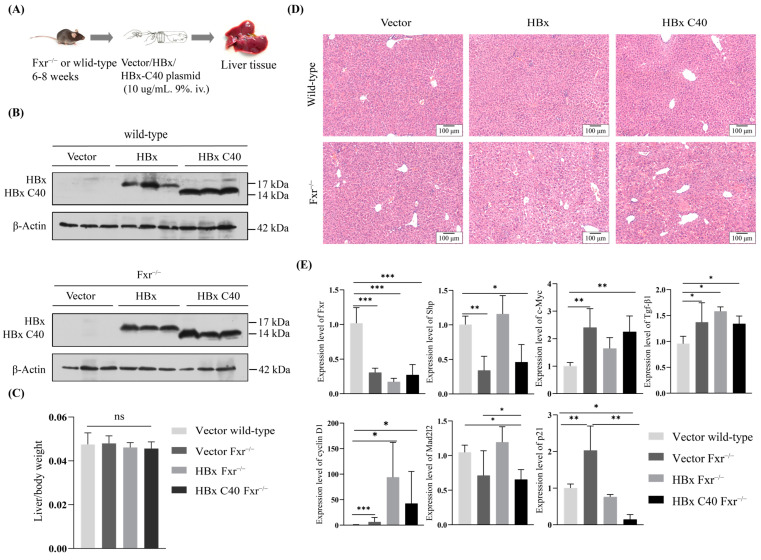
Effect of HBx or HBx C40 on transient mouse models. (**A**) Mouse model scheme of HBx C40 transient overexpression by hydrodynamic gene delivery. (**B**) Protein expression of HBx, and HBx C40 24 h after hydrodynamic gene delivery in wild-type or Fxr^−/−^ mice. (**C**) Ratio of the liver to body weight. (**D**) Representative images of H&E staining of mouse liver in each group are shown. Scale bar = 100 μm. (**E**) The mRNA expression of Fxr, Shp, c-Myc, Tgf-β1, cyclin D1, Mad2l2, and p21. * *p* < 0.05, ** *p* < 0.01, *** *p* < 0.001, ^ns^ no significance.

To determine the impact of HBx C40 in vivo, we first transiently overexpressed HBx C40 in wild-type and Fxr^−/−^ mice by hydrodynamic gene delivery. Wild-type and Fxr^−/−^ mice were injected with the empty vector plasmid as a control (Figure 4A). Protein expression of HBx and HBx C40 were determined by western blotting (Figure 4B). Although there was no obvious difference in the ratio of liver weight to body weight in mice (Figure 4C), microscopic hepatocytes from HBx C40 + Fxr^−/−^ mice showed slight alteration compared to Fxr^−/−^ hepatocytes in histomorphology (Figure 4D). We also detected the mRNA expression of Fxr, Shp, c-Myc, Tgf-β1, cyclin D1, Mad2l2, and p21 by qRT-PCR. The expression of Shp and p21 was downregulated in the Fxr^−/−^ group and HBx C40 + Fxr^−/−^ group, while c-Myc and cyclin D1 expression were elevated (Figure 4E).

### 2.5. HBx C40 Promotes Tumorigenesis in FXR KO Cells In Vivo

**Figure 5:**
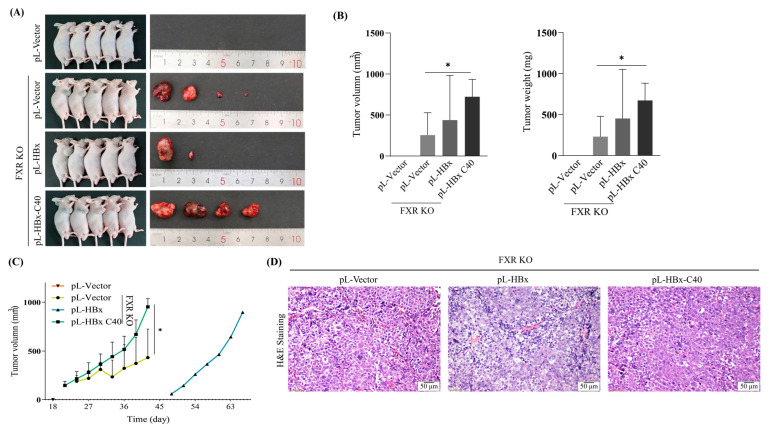
HBx C40 promotes tumorigenesis in FXR KO Hep3B cells in vivo. (**A**) Image of grafted tumors in nude mice following injection of NC, FXR KO, FXR KO + HBx, and FXR KO + HBx C40 Hep3B cells. (**B**,**C**) Tumor growth of each group of mice was recorded every 3 days. (**D**) Representative H&E staining of tumors in the NC, FXR KO, FXR KO + HBx, and FXR KO + HBx C40 groups is shown. The data represent the mean of the sample. Scale bar = 50 μm. * *p* < 0.05.

To further elucidate the oncogenic effect of HBx C40 in vivo, FXR KO Hep3B cells overexpressing HBx or HBx C40 were subcutaneously inoculated into male nude mice to generate hepatocellular carcinoma xenograft tumors. Results showed that the FXR KO cells had enhanced cell proliferation and tumorigenic ability in vivo compared to the control (Figure 5A), the final tumor size, weight, and tumor growth of HBx C40 overexpressing FXR KO cells were greater than those of the FXR KO control (Figure 5B,C). Additionally, the tumor hepatocytes had basophilic cytoplasm, a high nucleus/plasma ratio, a large number of necrotic areas of different sizes, and increased ability of angiogenesis, as judged by H&E staining, we failed to show the significant differences between them (Figure 5D). These data suggest that HBx C40 promotes cell proliferation and tumorigenicity capacity of FXR KO HCC cells in vitro as well as in vivo.

### 2.6. RNA Sequencing Identifies the Glucose Metabolism Signaling Pathway and Corresponding Crucial Genes in HBx C40-Expressing FXR KO Hep3B Cells

**Figure 6:**
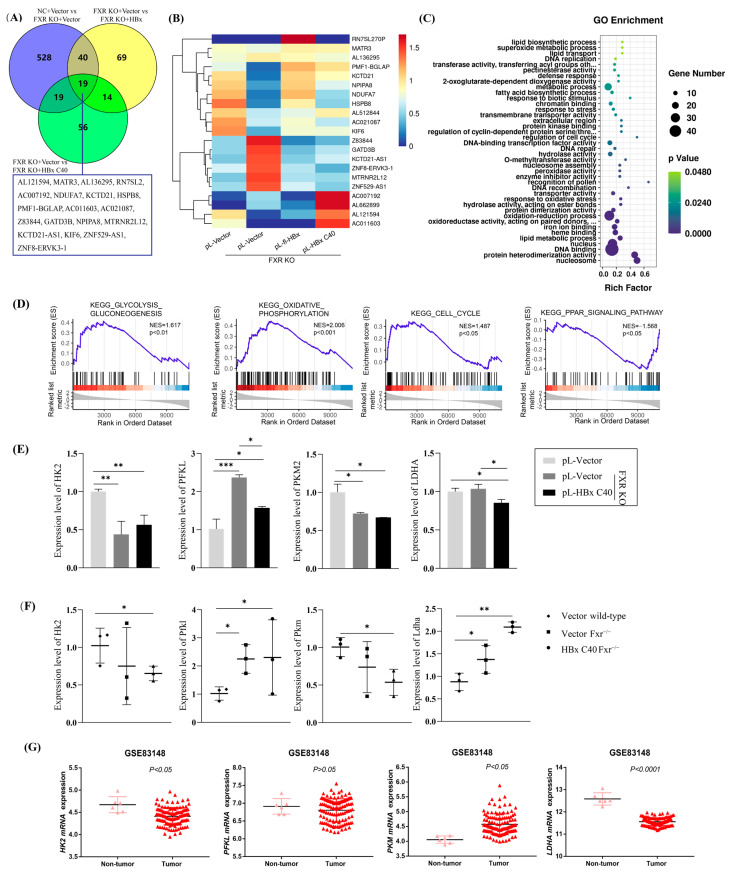
RNA sequencing identifies signaling pathways and crucial targets in HBx C40-expressing, FXR-deficient Hep3B cells. (**A**) DEGs are common among the NC vs. FXR KO Hep3B, FXR KO vs. FXR KO + HBx Hep3B, and FXR KO + HBx vs. FXR KO + HBx C40 Hep3B groups. (**B**) Heatmap of overlapping DEGs among the three groups. (**C**) GO signaling pathway enrichment. (**D**) Gene set enrichment analysis (GSEA). (**E**,**F**) The corresponding crucial genes expression levels in glucose metabolism were confirmed by qRT-PCR in vitro and in vivo. (**G**) Expression levels of crucial genes in tumor and non-tumor tissues in GSE83148 database. * *p* < 0.05, ** *p* < 0.01, *** *p* < 0.001.

Having shown that HBx C40 promotes tumor growth and to further explore the molecular mechanisms of HBx C40, we performed transcriptome sequencing to compare the gene profiles of NC, FXR KO, FXR KO + HBx, FXR KO + HBx C40 Hep3B cells. The threshold for differential gene screening was |log2 fold change| ≥ 1 and *p* < 0.05. Nineteen genes expressed differentially among all groups were overlapped (Figure 6A,B). GO analysis revealed that the molecular mechanisms involved in HBx C40-induced changes were closely related to cellular lipid metabolism, metabolic processes, cell cycle regulation, and oxidative stress (Figure 6C). Gene set enrichment analysis (GSEA) revealed that HBx C40 was actively involved in the regulation of glycolysis and gluconeogenesis, oxidative phosphorylation, and cycling under conditions of FXR deficiency (Figure 6D).

Numerous enzymes involved in glucose metabolism also play a role in tumor development and progression. We examined HK2, a key enzyme in glucose metabolism, and found that HK2 expression was downregulated by HBx C40 in FXR deficiency. To further solidify the roles of HBx C40, we also determined the expression profiles of crucial genes in glucose metabolism by qRT-PCR in vitro (Figure 6E) and in vivo (Figure 6F). In addition, the expression profiles of crucial genes in tumor and non-tumor tissues were verified in the HBV-positive GSE83148 database (Figure 6G).

### 2.7. HBx/HBx C40-HSPB8-HK2-Glucose Metabolism Axis in FXR-Deficient HCC

**Figure 7:**
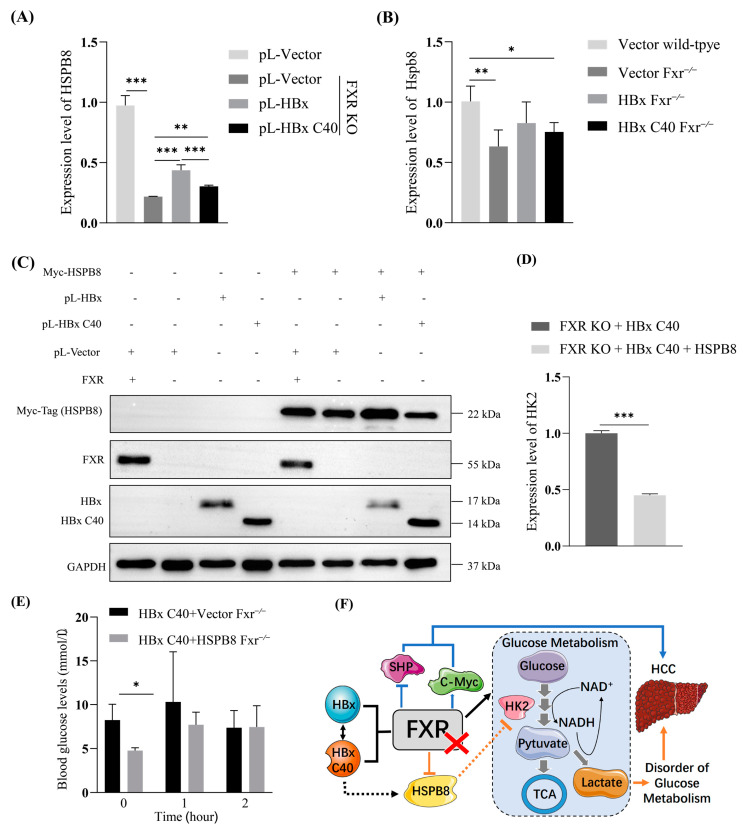
HBx/HBx C40-HSPB8-HK2-glucose metabolism axis in FXR-deficient HCC. (**A**,**B**) Differentially HSPB8 expression in cells and transient mouse models. (**C**) The overexpression of HSPB8 protein was detected by western blotting. (**D**) The mRNA expression of HK2 in HSPB8 + HBx C40 + FXR KO, and HBx C40 + FXR KO Hep3B cells were detected by qRT-PCR. (**E**) Fasting blood glucose, blood glucose 1 h after intraperitoneal injection of high glucose and 2-h postprandial blood glucose in transient mouse models. (**F**) Schematic diagram illustrating the induction of hepatocarcinogenesis, by HBx C40 expression combined with FXR deficiency, through inhibiting the regulation of HSPB8-HK2-glucose metabolism, including the gluconeogenesis and glycolysis axis, in the liver. * *p* < 0.05, ** *p* < 0.01, *** *p* < 0.001.

We noticed that HSPB8 was differentially expressed in our system, and has been reported to be involved in the regulation of a variety of tumors (Figure 7A). HSPB8 expression was down-regulated in FXR-deficient cells, whereas both HBx and HBx C40 increased HSPB8 expression. By contrast, HBx C40 would slightly reduce this increase (Figure 7B). To verify the effect of HSPB8 on the induction of hepatocarcinogenesis by HBx C40 in the absence of FXR, we overexpressed HSPB8 in HBx C40-overexpressing FXR KO Hep3B cells (Figure 7C). The expression profiles of glycolysis related genes were examined by qRT-PCR. HBx C40 overexpressing FXR KO Hep3B cells line had lower expression of HK2 when overexpressing HSPB8 (Figure 7D). Meanwhile, HSPB8 appeared to reduce fasting plasma glucose levels in Fxr^−/−^ transient model mice overexpressing HBx C40. One hour after the intraperitoneal injection of high glucose (2 g/kg), the blood glucose level did not change significantly (Figure 7E). Overall, our findings suggest that the hepatocarcinogenesis induction of HBx C40 could possibly be related to the dis-regulation of HBx/HBx C40-HSPB8-HK2-glucose metabolisms including gluconeogenesis and glycolysis axis (Figure 7F).

## 3. Discussion

Full-length HBx has been shown to protect against spontaneous hepatocarcinogenesis in mouse models by increasing FXR signaling [21]. Several different C-terminal truncations of HBx have been found in clinical liver tissue samples [6,12,13]. Furthermore, C-terminal truncated HBx has been shown to initiate hepatocarcinogenesis by downregulating TXNIP and reprogramming glucose metabolism [8]. FXR signaling plays a crucial role in maintaining normal liver function. Fxr^−/−^ mice are closely associated with high-fat diet-induced obesity, insulin resistance, and metabolic dysfunction-associated fatty liver disease (MAFLD) [13]. Liver-specific Fxr^−/−^ mice have abnormal glucose metabolism and severe fatty liver phenotypes. It is noteworthy that a low or deficient FXR expression is mostly associated with poor prognosis in mid- and late-stage HBV-infected HCC [16,27]. Due to the C-terminal truncation of HBx often occurring during the integration of HBV into the human genome [30], previous studies have found that although full-length HBx and C-terminal truncated HBx can be detected in tumor liver tissues, the function of the C-terminal truncated HBx is different from the full-length HBx [6,27]. Truncated HBx is more aggressive in promoting the development of HCC [10].

Here, our bioinformatic analysis shows that low FXR-expressing HCC patients have significantly shorter overall and progression-free survival and worse prognosis. Our in vivo FXR KO Hep3B cells tumorigenesis data suggest that FXR has an inhibitory effect on liver tumorigenesis. Fxr^−/−^ mice (C57BL/6-Nr1h4^em1cyagen^) in our laboratory can spontaneously develop tumors at 15 months, which may be associated with abnormal liver cholestasis and metabolic function. Our tumorigenesis experiments of FXR KO Hep3B cells confirmed that FXR KO Hep3B cells are highly tumorigenic, and FXR activation by CDCA can inhibit tumor growth.

C-terminal truncated HBx has been reported to be associated with cell proliferation, invasion, and metastasis [31], and also promotes hepatocarcinogenesis through regulation of cell cycling and apoptosis. HBx C40 overexpression, compared to HBx overexpression, in FXR KO HepB3 cells, promoted hepatocyte proliferation associated with upregulation of cyclin D1, inhibition of P21 expression, and alteration of cell cycle distribution. In addition, HBx C40 in FXR-deficient cells can reduce cleaved caspase-3 levels and upregulate the expression of c-Myc to reduce apoptosis. Together with the fact that HBx C40 induces cell cycle arrest in the G2/M phase and promotes FXR KO cell migration, these results suggest that HBx inhibits cell proliferation and promotes apoptosis in FXR-deficient cells. We also showed that HBx C40 enhances tumor formation in FXR-deficient cells in vivo. In addition, the expressions of c-Myc and cyclin D1 were elevated, whereas the expression of Shp and p21 were decreased in liver tissues, of Fxr^−/−^ mice, transiently overexpressing HBx C40. This suggests that in FXR deficiency, HBx C40 affects the biological functions of hepatocytes by altering the expression of c-Myc, Shp, cyclin D1, and p21 in mouse hepatocytes. All of the above suggest that HBx C40 plays an important role in promoting liver tumor growth in FXR deficiency.

The biological characteristics of tumors are often closely related to energy metabolism [32]. Our RNA-Seq analysis of FXR KO hepatocytes overexpressing HBx C40 showed that the different signaling pathways affected by HBx proteins are jointly involved in lipid metabolism and oxidative phosphorylation. Altered energy metabolic processes provide a viable microenvironment for tumors to meet the demands of rapid cancer cell growth. Numerous enzymes involved in glucose metabolism also play a role in tumor progression, including glucose-6-phosphate isomerase, which can inhibit apoptosis and promote tumorigenesis through PI3K/AKT activation. Isocitrate dehydrogenase 2 (IDH2) reduces HCC metastasis via matrix metallopeptidase 9 (MMP9) [33], and stearoyl-CoA-desaturase 1 (SCD1) regulates modulation of P53, Wnt/β-catenin, and autophagy in HCC [34,35,36]. FXR itself is involved in the regulation of gluconeogenesis and lipid metabolism. FXR can inhibit the expression of Phosphoenolpyruvate carboxykinase (PEPCK) and Glucose-6-Phosphate Isomerase (G6PI) in the hepatic gluconeogenic pathway. FXR deficiency decreases the inhibition of gluconeogenesis as well as lipogenesis and can cause an increase in de novo synthesis of fatty acids [37]. As a tumor suppressor gene, SHP is involved in glucose and lipid metabolism together with FXR [38,39]. The proto-oncogene c-Myc plays an important role in tumor cell proliferation and energy metabolism, and can be downregulated by FXR activation [40,41]. Our data suggest that FXR deficiency down-regulates SHP and up-regulates c-Myc, thereby promoting HCC progression. C-terminal truncated HBx has been shown to promote carcinogenesis by interfering with glycolytic reprogramming [8]. We observed that when FXR is deficient, HBx C40 can affect the expression of key enzymes in glycolysis, such as downregulating HK2, the rate-limiting enzyme responsible for the catalytic conversion of glucose to glucose-6-phosphatase (G-6-P), initiating glycolysis [42]. High expression of HK2 in HCC tissues is closely associated with patient poor prognosis [43]. HK2 can bind to voltage-dependent anion channel 1 (VDAC1), which increases ATP and inhibits apoptosis [44], and also reduces downstream G6PI inhibition by VDAC1 thereby enhancing glycolytic [45]. HSPB8 can also be involved in tumorigenesis [26]. HBx C40 upregulated the expression of HSPB8 in FXR deficiency. Overexpression of HSPB8 in FXR KO Hep3B showed that HSPB8 could downregulate the expression of HK2 and others and exacerbate the disruption of glucose metabolism.

In summary, we found that HBx C40 could promote cell proliferation and migration and alter the hepatocyte cell cycle under conditions of FXR deficiency. Moreover, we showed that the induction of hepatocarcinogenesis by overexpressing HBx C40, in the absence of FXR, is associated with an HBx C40-HSPB8-HK2 axis that disturbs the liver cellular energy metabolisms, including gluconeogenesis and glycolysis. These findings may help us to better understand the molecular mechanisms of C-terminal truncated HBx in HBV infection-mediated tumorigenesis.

## 4. Materials and Methods

### 4.1. Cell Culture

The human Hep3B hepatocellular carcinoma cell line Hep3B was obtained from the Cell Bank of the Chinese Academy of Sciences and maintained in Dulbecco’s modified Eagle’s medium (DMEM; Gibco, New York, NY, USA) supplemented with 10% fetal bovine serum (FBS; Gibco, New York, NY, USA), 1% penicillin-streptomycin solution (Beyotime, Shanghai, China), and 1% non-essential amino acids (NEAA, 100×; Gibco, New York, NY, USA). All cells were cultured at 37 °C with 5% CO_2_ in a humidified incubator.

### 4.2. Plasmids

The expression plasmids and vectors for Flag-tagged HBx, and HBx C40 have been previously described [21,46]. The Myc-tagged HSPB8 plasmid was purchased from Origene (MD, USA). Lentivirus with full-length HBx (Myc-tagged HBx), C-terminal truncated HBx (Myc-tagged HBx C40), and empty lentiviral vector (Plenti-CMV-Myc-PGK-EGFP-F2A-Puro-WPRE) were constructed by and purchased from OBiO Technology Co., Ltd. (Shanghai, China).

### 4.3. Transient Transfection

According to the manufacturer’s protocol, Lipofectamine 2000 reagent (Thermo Fisher Scientific, Carlsbad, CA, USA) was used for plasmid transfection. At 24 or 48 h after transfection, the cells were harvested for further experiments.

### 4.4. Stable Transfection, Lentiviral Production, and Cell Transduction

A stable FXR knockout (FXR KO)/negative control (NC) model in the Hep3B cell line was created using the CRISPR-U™ system (Ubigene, Guangzhou, China). The sequences of the gRNA Sequence targeting FXR were as follows: FXR gRNA #1 (CCTGAAGAGTGGTACTCTCCTGG), FXR gRNA #2 (TGCATTATAGTG GTATCCAGAGG). Plasmids were transfected into FXR KO/NC Hep3B cell lines followed by selection with puromycin (Beyotime, Shanghai, China) at 1 μg/mL for one week. Cells were infected with a lentiviral vector as previously described [46], and the expression of HBx or HBx C40 was detected by qRT-PCR and western blotting.

### 4.5. RNA Extraction and Quantitative Real-Time Polymerase Chain Reaction (qRT-PCR)

Total RNA from cells and tissues was extracted with TRIzol^®^ (Invitrogen, Carlsbad, CA, USA), and 1 µg of total RNA was reverse transcribed using a PrimeScript RT reagent kit with gDNA Eraser (Perfect Real Time; Takara, Tokyo, Japan) based on the manufacturer’s recommendations. qRT-PCR was performed on the QuantStudio 5 Real-Time PCR System (ABI, Marsiling Industrial Estate, SGP) by using Takara TB Green Premix Ex Taq (TaKaRa, Tokyo, Japan) and analyzed using the 2^−∆∆Ct^ method and normalized to cyclophilin. The qRT-PCR primer sequences are presented in Table 1.

### 4.6. Cell Viability and Colony Formation Assay

Cell viability was assessed with a Cell Counting Kit-8 (CCK-8; MCE, Shanghai, China). After transfection, cells were seeded into the 96-well plates at a density of 2 × 10^3^ cells/well in 100 µL DMEM with 10% FBS, and the plates were incubated for 24, 48, 72, and 96 h. Then, the cells were incubated with a 10% CCK-8 working solution for 2 h in the dark at 37 °C. The absorbance of cells at 450 nm was detected by a microplate reader. Infected cells were plated in 6-well plates for colony formation analysis at 500 cells/well and maintained for 15 days. For visualization of the colonies, cells were fixed with 4% paraformaldehyde for 20 min and stained with 0.1% crystal violet for 30 min. The colony numbers were then quantified.

### 4.7. Cell Migration Assay

Cell migration and invasion were assessed by wound healing and Transwell assays. In the wound healing assay, cells were plated in 6-well dishes and grown to 95% confluence. Then, three artificial straight scratched wounds were quickly produced on the monolayer of cells with a sterile 200 µL pipette tip. Observations were made using a microscope (magnification of 100×) at 0, 24, and 48 h.

### 4.8. Cell Cycle Analysis

Cell cycle analyses were performed by flow cytometry. At 85–90% confluence, the cells were collected and fixed in 70% pre-cooled ethanol at 4 °C for 24 h, and then stained with PI staining solution containing 100 µg/mL PI (MCE, Shanghai, China) and 50 µg/mL of RNase A (Solarbio, Beijing, China) for 30 min at 37 °C. Then the cell cycle distribution was detected by flow cytometry and analyzed using BD Accuri C6 Software.

### 4.9. Cell Apoptosis Analysis

FXR KO Hep3B cells infected with HBx or HBx C40-encoding lentivirus were plated into 6-well plates. After a 24 h incubation, cells were fixed with 4% paraformaldehyde for 30 min at RT. Next, cells were incubated with 0.3% Triton X-100 for 5 min at RT. Subsequently, the cells were incubated with a TUNEL assay working solution for 60 min at 37 °C according to the Colorimetric TUNEL Apoptosis Assay Kit’s (Beyotime, Shanghai, China) instructions, followed by the addition of Antifade Mounting Medium with DAPI solution and photographed under a microscope (magnification of 100×).

### 4.10. Western Blotting

Cells and tissues were lysed and sonicated in cold RIPA buffer (Beyotime, Shanghai, China) and tissue lysis both with protease inhibitor cocktail (Thermo Fisher Scientific, Carlsbad, CA, USA), respectively. Lysates were centrifuged at 4 °C for 15 min at 12,000 rpm, supernatant was collected, and the protein concentration was determined using a bicinchoninic acid (BCA) Protein Assay Kit (Invitrogen, Waltham, MA, USA). Equal amounts of proteins were run on 10–15% sodium dodecyl sulfate-polyacrylamide gel electrophoresis (SDS-PAGE) and transferred to 0.22 μm polyvinylidene difluoride (PVDF) membranes (Merck Millipore, Tullgren, Carrigtwohill, Co., Cork, Ireland). Then, the PVDF membranes were blocked using 5% nonfat milk at RT for 2 h and incubated with primary antibody (FXR, A9033A, R&D; GAPDH, BM1985, BOSTER; ab39716, Abcam; Bcl-2, 40639, SAB; cleaved caspase-3, 29034, SAB) at 4 °C overnight. Subsequently, after probing with HRP-conjugated secondary antibodies for 2 h at RT, protein bands were detected with SuperSignal West Pico PLUS chemiluminescence substrate (Invitrogen, Waltham, MA, USA).

### 4.11. Subcutaneous Nude Murine Xenograft Models

All animal experiments were carried out with the permission of the Shantou Medical Experimental Animal Care Commission and approved by the Institutional Animal Care and Research Advisory Committee of Shantou University Medical College (SUMC2017-029). Male BALB/c nude mice, 4–6 weeks old, were purchased from the Beijing Vital River Laboratory Animal Technology and maintained under specific pathogen-free (SPF) conditions. Mice were subcutaneously injected with 2 × 10^6^ of Hep 3B cells. NC and FXR KO cells were injected on the right and left sides, respectively. After 24 days, when the tumor volume had reached 50 mm^3^. The mice were randomly divided into two groups and gavaged with 200 µL of vehicle or 0.5% CDCA per day. Growth of the transplanted tumor was measured every three days. Tumor volume (V) was monitored by measuring the length (L) and width (W) with calipers and calculated using the formula: V = (L × W^2^)/2.

In experiments comparing tumor growth of the different cell lines, 2 × 10^6^ of NC + Vec, FXR KO + Vec, FXR KO + HBx, or FXR KO + HBx C40 Hep3B cells were injected subcutaneously into nude mice, respectively. Tumor size and weight were monitored every 3 days.

### 4.12. Transgenic Fxr^−/−^ Mice and Hydrodynamic Liver Transfection and Blood Glucose Assay

C57BL/6-Nr1h4^em1cyagen^ (Fxr^+/−^) mice were obtained from Cyagen (Santa Clara, CA, USA), and Fxr^−/−^ and wild-type mice were generated by breeding in our laboratory. Male mice, 6–8 weeks old, received hydrodynamic tail vein injection, at 10% of body weight per mouse, with a sterile PBS solution containing 10 µg/mL of the plasmid [21]. The injection was done quickly and uniformly taking no longer than 4 to 7 s. Twenty-four hours post-injection, mice were euthanized by cervical dislocation and livers were harvested after cardiac puncture and aspiration. Then tissues were collected for paraffin embedding and other experiments.

In another experiment, Fxr^−/−^ mice were fasted for 24 h and were allowed to drink freely. At 6 h after injection of HBx C40 + vector and HBx C40 + HSPB8 plasmid, basal blood glucose levels were measured from tail bleeds by glucometer (Sinocare, China) and free diet was resumed. Then 50% hyperglycemic solution (0.0067 mL/g) was injected intraperitoneally to measure blood glucose levels 1 h of high glucose and 2-h postprandial blood glucose.

### 4.13. Gene Expression Profiling and Functional Analysis

Transcriptome analysis was performed in NC + Vec, FXR KO + Vec, FXR KO + HBx, and FXR KO + HBx C40 Hep3B cells were obtained as described previously [47]. Total RNA was extracted using TRIzol according to the manufacturer’s instructions. Paired-end sequencing of the library in PE150 mode was performed on an Illumina NovaSeq 6000 platform by LC-Bio Technologies (Hangzhou, China) Co., Ltd. The transcript and expression levels of the samples were then quantified using StringTie to calculate FPKM (fragments per kilobase million) values, with |log2 Fold Change| ≥ 1 and false discovery rate (FDR) *p* < 0.05 as cut-off criteria for DEGs. DEGs, GO analysis, and KEGG enrichment analyses were performed using the OmicStudio tools (https://www.omicstudio.cn/tool) accessed on 2 April 2022. The identified DEGs containing above-mentioned parameters were validated by qRT-PCR.

### 4.14. Hematoxylin and Eosin (H&E) Staining

The collected tumors or mouse liver tissues were fixed in a 4% paraformaldehyde solution for 24 h and then observed under the microscope after tissue fixation, paraffin embedding, sectioning, and hematoxylin/eosin staining.

### 4.15. Statistical Analysis

Student’s *t*-test, one-way ANOVA, or two-way repeated-measures ANOVA were applied. Statistical significance was assessed by comparing mean values ± SD. All statistical analyses involved using SPSS 19.0 (SPSS Inc., Chicago, IL, USA). Statistical significance was indicated by * *p* < 0.05, ** *p* < 0.01, and *** *p* < 0.001.

## 5. Conclusions

HBx C40 facilitates oncogenesis by modulating cell cycle and glucose metabolism in FXR-deficient hepatocellular carcinoma.

## Figures and Tables

**Table 1:** List of primers used for quantitative real-time PCR analysis.

Gene	Forward (5′-3′)	Reverse (5′-3′)
hFXR	GCATTACCAAAAACGCTGTG	CAGCCAACATTCCCATCTCT
hSHP	GAATATGCCTGCCTGAAAGG	TCCAGGACTTCACACAGCAC
hCYP7A1	GAGAAGGCAAACGGGTGAAC	GCACAACACCTTATGGTATGACA
hCyclophilin	TGGTGTTTGGCAAAGTGAAA	TCGAGTTGTCCACAGTCAGC
hTGF-β1	CTAATGGTGGAAACCCACAAGG	TATCGCCAGGAATTGTTGCT
hc-Myc	CACTAACATCCCACGCTCTGA	AAACCGCATCCTTGTCCTGT
hCyclin D1	GATCAAGTGTGACCCGGACTG	CCTTGGGGTCCATGTTCTGC
hP21	AGTCAGTTCCTTGTGGAGCC	CATTAGCGCATCACAGTCGC
hHSPB8	AAGCCAGAGGAGTTGATGGTG	CTCTGCAGGAAGCTGGATTTT
hHK2	TCGCCGGTAGCCTTCTTTGT	AGAGATACTGGTCAACCTTCTGC
hPFKL	CGGTGGACCTGGAGAAGCTG	TCCAGGCGGAGTCAATGTG
hPKM2	ATGCAGCACCTGATAGCTCG	GTGGAGTGACTTGAGGCTCG
hLDHA	TTGTCTCTGGCAAAGTGGATATCTT	CCACTCCATACAGGCACACT
mCyclophilin	GGCTGAGAACGGGAAGCTTGTCAT	CAGCCTTCTCCATGGTGGTGAAGA
mFxr	TCGTTCGGCGGAGATTTTCA	TGTAGCACATCAAGCAGGGG
mShp	TCCTCTTCAACCCAGATGTGC	TCTCCCATGATAGGGCGGAA
mc-Myc	GTTGGAAACCCCGCAGACAG	ATAGGGCTGTACGGAGTCGT
mcyclin D1	AGAACAAGCAGACCATCCGC	GTCCTTGTTTAGCCAGAGGC
mp21	GCAGAATAAAAGGTGCCACAGG	AAAGTTCCACCGTTCTCGGG
mMad2l2	TCCACTGCGTCAAACCTCTC	TAAATGTGCAGCCTGGAGGG
mHk2	AAGCTTCTTTGTGTGGCTCCT	AGAGATACTGGTCAACCTTCTGC
mPfkl	GGCTCTCGGCTGAACATCAT	TGTGGTTCTGGAGGCATCCTT
mPgk1	CCTCTCCTTCCTTTTAGACGCC	AGTCTTCCTCCAGTTCGCTCA
mPkm	GCAGCGACTCGTCTTCACT	GCATGGTTCCTGAAGTCCTCG
mLdha	AACTTGGCGCTCTACTTGCT	GGACTTTGAATCTTTTGAGACCTTG
mHspb8	CAATTGCCTTTCCCGTGCTC	TCTGGAAAAGGGTCCATGCC

h, Human; m, Mouse.

## Data Availability

Not applicable.
